# Wide QRS-T angle and low T wave amplitude are associated with the presence of myocardial expansion as measured by extracellular volume fraction with cardiac MRI

**DOI:** 10.1186/1532-429X-18-S1-P275

**Published:** 2016-01-27

**Authors:** Karolina M Zareba, Akira Wada, Xiaojuan Xia, Jean-Philippe Couderc, Subha V Raman

**Affiliations:** 1 Ohio State University, Columbus, OH USA; 2 University of Rochester, Rochester, NY USA

## Background

Extracellular matrix expansion as measured by extracellular volume fraction (ECV) with cardiac MRI (CMR) is a strong predictor of clinical outcomes. The aim of our study was to evaluate the relationship between EKG parameters and the presence of myocardial expansion as measured by ECV.

## Methods

We studied 90 consecutive patients without myocardial infarctions (mean age 53 years, 50% female) referred for CMR with T1 mapping that had a 12 lead EKG within 90 days. Numerous EKG parameters were evaluated visually and electronically (heart rate, PR duration, QRS amplitude and duration, QRS morphology, QRS-T angle, QRS fragmentation, T wave amplitude, QTc duration, corrected QT peak duration, T peak T end) to determine their association with myocardial fibrosis as measured by ECV.

## Results

There were 40 (44%) patients with an elevated ECV ≥ 29%. Patients with an elevated ECV were more likely to have a history of congestive heart failure (63% vs 16%, p = 0.000), had lower ejection fraction (44% vs 56%, p = 0.000), and had more non-ischemic scar (63% vs 24%, p = 0.000) as noted with late gadolinium enhancement (LGE). In patients with an elevated ECV, QRS Cornell amplitude (1.7 vs. 1.2 mV, p = 0.018), and QRS-T angle (112° vs. 57°, p = 0.000) were significantly higher, while T wave amplitude (45 vs. 229 µV, p = 0.002) was significantly lower. Multivariate logistic regression revealed that QRS-T angle ≥100° (OR 4.0, p = 0.015) and T wave amplitude ≤ 170 µV (OR 4.2, p = 0.011) were highly associated with an elevated ECV after adjustment for clinical covariates (heart failure, ejection fraction, LGE, bundle branch block).

## Conclusions

Wide QRS-T angle and low T wave amplitude were independently associated with the presence of myocardial expansion as measured by ECV. Prospective evaluation of these parameters can aid in appropriate CMR referral for patients at risk of myocardial disease.

